# Parents' concerns about children are highly prevalent but often not confirmed by child doctors and nurses

**DOI:** 10.1186/1471-2458-8-124

**Published:** 2008-04-18

**Authors:** Sijmen A Reijneveld, Gea de Meer, Carin H Wiefferink, Matty R Crone

**Affiliations:** 1 University Medical Center Groningen, University of Groningen, Department of Health Sciences/SHARE, PO Box 196, 9700 AD Groningen, The Netherlands; 2 TNO (Netherlands Organization of Applied Scientific Research) Quality of Life, Division of Child Health, Leiden, The Netherlands

## Abstract

**Background:**

The aim of this study was to assess the prevalence in the general population of parents' concerns about the development of their child, to identify groups at risk and to assess the association between parents' concerns and professional judgement.

**Methods:**

We obtained cross-sectional data on a Dutch nationally representative sample of children aged 14 months, 3 3/4, 5–6 and 8–12 years within the setting of routine well-child visits provided to the entire population. A total of 4,107 participated (response rate 85.3%). Data were about concerns that parents reported by questionnaire before the visit regarding behavioural and emotional problems, developmental delay, consequences of disease and contact with peers that needed professional assistance, and about the assessment of these domains by doctors and nurses during the visit. Moreover, we obtained data on parent-reported psychosocial problems using the Infant-Toddler Social and Emotional Assessment and the Child Behavior Checklist.

**Results:**

Of all parents, 49.3% reported some concerns and 8.7% reported frequent concerns, most frequently on child behaviour. Frequent concerns were most likely to refer to young children, children from labour immigrant families, with fathers of medium educational level and in low-income families. The prevalence rates of professional-assessed parenting problems were much lower than parent-reported ones. The rates of psychosocial problems were highest in the case of shared concerns, but also higher if parents expressed concerns that were not confirmed by professionals.

**Conclusion:**

A very large proportion of parents of young children have concerns regarding their child, but agreement on these concerns with child health professionals is relatively low.

## Background

Parents' concerns about their child are pivotal in seeking care and contacting health professionals. In clinical practice, the prevalence rate of concerns among parents will thus be very high, at least at first entry into care. In developmental surveillance during paediatric preventive care visits, a large majority of the US primary care physicians reports the use of eliciting parental concerns on development as a tool [1].

In contrast, little is known about the prevalence of parental concerns in the community, their nature, or their distribution across various groups. Much of the little that is known comes from the work by Glascoe and co-workers [2-4]. Her work reveals that these concerns occur frequently among US parents and that in particular serious concerns reported in standardized questions may give a rather accurate indication of the need to refer a child [3]. A summary of four studies on the prevalence rates of the various types of concerns in the community yields a prevalence rate of 45% [5]. Ford and co-workers do provide prevalence rates for concerns among the parents of 10,438 British children aged 5–15. In their study, 9.5% of all parents report at least one concern regarding behaviour, emotion or activity level; the figures are 5.5% and 4.1% for behaviour and emotion, respectively. This lower prevalence may be explained by the fact that they focussed on the child having a problem in the opinion of the parent, which may be more confined than 'concern'. The sensitivity for a psychiatric disorder of at least one concern about a psychiatric disorder is 47%, i.e. 47% of the children with concerned parents have any psychiatric disorder; the specificity was 94% [6]. Finally, Blanchard et al. report on the prevalence of parental concerns regarding a range of developmental areas in a sample of 102,353 US children. They show that parental concerns about specific domains are highly prevalent, for instance 41% for learning difficulties and 36% for depression and anxiety in children aged 6–17. No evidence is available in the literature on the prevalence of concerns by family and child background.

Preventive child healthcare, i.e. well-baby and well-child visits, offers an ideal opportunity to assess parents' concerns and to arrange care if needed. In the Netherlands, this system has been set up just for prevention and screening, including the provision of the national vaccination programme, and it is separate from other child health treatment services, and with access independent of insurance status. Contacts occur in a setting other than where medical services are provided [7,8]. Contact rates are very high; measured by vaccination rates for diphtheria, tetanus and poliomyelitis in 2005, they varied from 97.8% for infants to 95.2% for children aged 6 and 95.1% for those aged 12 (for the latter age group, rates for other infectious diseases like measles were even higher, 97.7%) [9]. Because of this demarcation, it is an excellent route to obtain population-based figures on the occurrence of parents' concerns.

The aim of this study was to assess the prevalence of parents' concerns about various aspects of their children, to assess groups with a higher occurrence and to assess the association between parents' concerns and professional judgement.

## Methods

We collected data within the framework of the routine preventive health assessments that are provided regularly to all Dutch children from October 2002 until May 2003. The study was approved by the local Medical Ethical Committee and included verbal informed consent by the parents.

### Participants

We obtained a national sample using a two-stage selection procedure. In the first stage, two random samples of Dutch Child Healthcare Services were drawn using random numbers, one out of those providing services for preschool children (i.e. under age 4 in the Netherlands; 10 out of a total of 60 services), and one for school-aged children (15 out of a total of 40 services) [10]. Both samples were stratified by region and degree of urbanization of their district. In the second stage, each service provided a random sample of about 100 children for four age bands as far as they provided services for them (14 months; 3 years and 9 months; 5–6 years; and 8–12 years). Variation allowed for the two youngest groups were +/- 2 and 3 months, respectively. The oldest two groups concerned grades 2 and 5–8 of Dutch primary school, respectively. Common ages for the two oldest groups were 5–6 years and 8–12 years, respectively (but groups were composed based on grade). This yielded a sample of 4,776 children (response rate 85.3%). Differences between responding and non-responding families by sex, age, ethnic background and degree of urbanization were small, according to Cohen effect size w (range of w, 0.006–0.167), being largest for urbanization (all p > 0.05). Sampling was organized similarly to that in previously reported studies on the Dutch preventive child healthcare system [7,8].

### Measures and procedures

Data were obtained during routine well-child visits. Prior to the visit, parents received an invitation by mail to participate in the study, accompanied by a questionnaire on, *inter alia*, their concerns regarding parenting. The questionnaires were returned at the visit before the assessment, in sealed envelopes; they were not inspected. The doctor or nurse working in preventive child healthcare (henceforth: CHP, child health professional) then performed a routine preventive health assessment, consisting of an interview and an assessment that covered a routine list of topics for each age group, and filled out pre-coded questions on the development of their child (see below).

Data on *parents' concerns *were obtained by questions in the questionnaire to be filled out by parents on the occurrence of concerns during the past 12 months regarding parenting in general, developmental delay, behaviour, emotions, consequences of diseases, and contact with peers for which they felt that they needed assistance or advice from someone outside the family. This question was reported for each of the domains mentioned. Regarding the concerns in each domain, parents could answer 'no concerns', 'some concerns' or 'frequent concerns'. If they had concerns, parents were also asked whether they had looked for professional help, and if not, why not.

Parent-reported *behavioural and emotional problems *were assessed using the validated Dutch 2001 version of the Child Behavior Checklist (CBCL) for children aged 3 and over, using the version for the appropriate age group [11,12]. This was also included in the questionnaire to be filled out before the interview. Dutch, English, Turkish and Arabic versions were available, depending on the registered country of birth of the parents. For children aged 14 months we used the validated Dutch version of the Infant Toddler Social and Emotional Assessment (ITSEA) [13,14]. Both the CBCL and the ITSEA are reliable and valid measures of behavioural and emotional problems over the preceding six months, used in various countries and cultures. The CBCL contains 100 to 120 problem items on the basis of which a Total Problems Score can be computed (the higher the score, the more problematic the child), as well as separate scores regarding behavioural and emotional problems designated as the Externalizing and Internalizing problems broad-bands, respectively [11,12,15-20]. From the ITSEA, we similarly used the Externalizing and Internalizing problems scale [21].

*CHP-identified parenting problems *concerned the response of the CHP to the question 'Do the parents have problems with parenting?' asked at the end of each visit, in a separate questionnaire that also concerned further findings made during the visit. If the answer was yes, the subsequent question concerned the cause of these problems (regarding the child: emotional problems, behavioural problems, developmental delay, (physical) disease of the child; regarding the parents: limited parenting skills).

Finally, CHPs registered data on the *background characteristics *of the child and its family: ethnic background, parental educational level, employment and age, family composition, family income, number of siblings. Ethnic background was assessed by country of birth of the child's parents. On the basis of the migration histories of various groups living in the Netherlands, this was coded as Dutch-born; from a (former) Dutch colony (at least one parent born in Surinam or the Dutch Antilles); from countries in which Dutch employers recruited unskilled labourers in the 1960s and 1970s ('labour immigrant', at least one parent born in Turkey or Morocco); other industrialized countries; and other non-industrialized countries [22]. The educational level of each parent concerned the highest degree obtained by that parent. Family composition focused on the number of parents in the family (biological father and mother, two parents but at least one non-biological, only one, and other situations such as two parents of the same gender). Family income was categorized as below or at poverty level vs. higher. All characteristics were registered by the CHP during the assessment. Categories of all variables are presented in Table 1.

**Table 1:** Prevalence rates of parental concerns regarding their child by domain and frequency (n = 4,107) *

	No concerns		Some concerns		Frequent concerns	
Behavioural problems	3078	75.4%	842	20.6%	162	4.0%
Emotional problems	3453	84.7%	527	12.9%	95	2.3%
Parenting in general	3234	79.2%	753	18.4%	98	2.4%
Developmental delay	3572	87.5%	449	11.0%	61	1.5%
Consequences of disease	3462	85.0%	504	12.4%	106	2.6%
Contact with peers	3683	90.3%	344	8.4%	50	1.2%

* Percentages do not always add up to 100% due to rounding off; not all parents filled out questions about all concerns

All CHPs received a half-day training session on the procedures to be followed. Training was given at the service by one or two members of the research team. During the data collection, they were contacted every four weeks to monitor the quality of the data collection.

### Analysis

We first assessed the prevalence rates of any parenting concerns, then separately those of frequent concerns, as well as the mean number of domains in which concerns are present. Next, we assessed differences by background characteristics. We selected background characteristics that were associated with concerns by multiple logistic regression using forward selection with p < 0.05 as the selection criterion. We repeated this for frequent concerns, and confirmed both analyses using backward selection procedures. We then assessed the degree to which frequent parental concerns were associated with professional judgement by using cross-tabulations with chi-square tests, and computing kappa [23]. Fourth, we looked at discrepancies between parent report and professional judgement. Regarding this we also looked at the parent-reported scores of children for the relevant outcomes that were available, i.e. clinical externalizing and internalizing problems on the CBCL and ITSEA, and parent-reported consequences of disease. Using the CBCL/ITSEA as the golden standard, we also computed sensitivity and specificity for any parental concern in the particular domain. Finally we assessed the reasons why parents with frequent concerns did not seek professional assistance. Analyses were limited to children with complete parent-reported and CHP-registered data (n = 4,107); differences between the overall sample and the analyzed group were as small as between the overall sample and the total group of respondents.

## Results

### Parents' concerns

**Figure 1:**
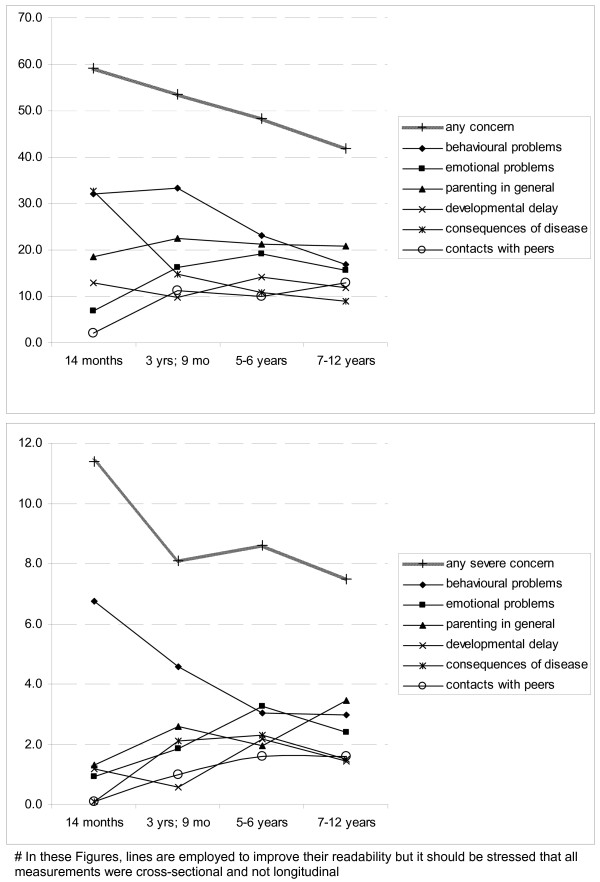
Prevalence rates of at least some concern (above) and of frequent concerns (below), by domain and child age.

Concerns of parents were highly prevalent with 49.3% of all parents reporting some concerns on at least one topic and 8.7% reporting frequent concerns on at least one topic. The highest prevalence rates applied to concerns about the behaviour of the child, see Table 1. Prevalence rates largely differed by age with those on behavioural problems and on consequences of disease in particular being lower when the child was older and most of the other ones being somewhat higher (Figure 1).

Prevalence rates varied largely by background characteristics, being especially high in one-parent families and in families with incomes below the poverty line, and also increased among single children, and children of young and medium- of high-educated fathers (Table 2). A logistic model on frequent concerns in any domain shows that (the group with the highest risk is between brackets) child age (young, i.e. 14 months), ethnic background (labour immigrant), father's educational level (medium or unknown), and family income (low) were independent predictors for the existence of frequent concerns (Table 3).

**Table 2:** Prevalence rates of parental concerns regarding their child, and number of domains about which they have any concern and frequent concerns, by background characteristics

	Prevalence rate of at least one concern			Number of domains on which: any concerns			frequent concerns		
			P-value	Mean	*SE*	P-value	Mean	*SE*	P-value

Gender									
-boy	978/2048	47.8%	0.051	0.92	*0.03*	0.018	0.12	*0.01*	0.046
-girl	1046/2059	50.8%		1.02	*0.03*		0.16	*0.01*	
Age of the child									
-14 months	450/760	59.2%	0.000	1.05	*0.04*	0.001	0.16	*0.02*	0.812
-3 years 9 months	377/703	53.6%		1.07	*0.05*		0.13	*0.02*	
-5–6 years	673/1392	48.3%		0.98	*0.04*		0.14	*0.01*	
-8–12 years	524/1252	41.9%		0.86	*0.04*		0.13	*0.02*	
Ethnicity									
-Dutch	1585/3232	49.0%	0.935	0.93	*0.02*	0.001	0.12	*0.01*	0.000
-former colony	81/162	50.0%		0.96	*0.10*		0.20	*0.05*	
-labour migrant	191/392	48.7%		1.14	*0.08*		0.27	*0.04*	
-other non-industrialized	110/208	52.9%		1.26	*0.11*		0.19	*0.05*	
-other industrialized	46/91	50.5%		1.02	*0.14*		0.15	*0.05*	
Family composition									
-two-parent	1800/3743	48.1%	0.000	0.93	*0.02*	0.000	0.13	*0.01*	0.000
-one-parent	184/310	59.4%		1.36	*0.08*		0.29	*0.05*	
-other	38/47	80.9%		1.57	*0.18*		0.26	*0.13*	
Employment status									
-unemployed	102/183	55.7%	0.089	1.22	*0.12*	0.007	0.27	*0.06*	0.002
-employed	1719/3534	48.6%		0.95	*0.02*		0.13	*0.01*	
-unknown	203/390	52.1%		1.05	*0.07*		0.17	*0.03*	
Maternal educational level									
-low	735/1563	47.0%	0.144	0.95	*0.03*	0.894	0.16	*0.01*	0.130
-medium	745/1482	50.3%		0.98	*0.03*		0.14	*0.01*	
-high	507/988	51.3%		0.98	*0.04*		0.11	*0.01*	
Paternal educational level									
-low	664/1407	47.2%	0.005	0.94	*0.03*	0.000	0.14	*0.01*	0.000
-medium	638/1289	49.5%		0.96	*0.04*		0.15	*0.02*	
-high	574/1162	49.4%		0.94	*0.04*		0.10	*0.01*	
-unknown	148/249	59.4%		1.36	*0.09*		0.31	*0.05*	
Family income									
-below poverty	257/428	60.0%	0.000	1.47	*0.08*	0.000	0.30	*0.04*	0.000
-above poverty	1532/3126	49.0%		0.93	*0.02*		0.12	*0.01*	
-unknown	235/553	42.5%		0.80	*0.05*		0.12	*0.02*	
Urbanization									
-rural/small city	1703/3448	49.4%	0.749	0.96	*0.02*	0.086	0.13	*0.01*	0.093
-big city	321/659	48.7%		1.05	*0.05*		0.17	*0.02*	
Number of siblings									
-no sibs	497/874	56.9%	0.000	1.10	*0.04*	0.000	0.13	*0.02*	0.943
-1 sib	980/1904	51.5%		1.04	*0.03*		0.14	*0.01*	
-2 and > sibs	547/1329	41.2%		0.80	*0.03*		0.14	*0.02*	
Maternal age									
-< 27	438/844	51.9%	0.173	1.10	*0.05*	0.002	0.17	*0.02*	0.016
-27–33	1124/2274	49.4%		0.96	*0.03*		0.15	*0.01*	
-33+	449/960	46.8%		0.88	*0.04*		0.09	*0.01*	
Paternal age									
-< 27	192/379	50.7%	0.043	1.13	*0.07*	0.000	0.17	*0.03*	0.009
-27–33	987/1959	50.4%		0.98	*0.03*		0.14	*0.01*	
-34–40	618/1326	46.6%		0.88	*0.03*		0.11	*0.01*	
-41+	140/293	47.8%		0.96	*0.08*		0.15	*0.03*	

**Table 3:** Background characteristics that are associated with frequent parenting concerns, after mutual adjustment: odds ratios (OR) and 95% confidence intervals (CI) (n = 4,107)

	Prevalence rate	OR	*95%-CI*		P-value
Ethnicity					
- Dutch	7.6%	1.00			0.007
- former colony	13.0%	1.50	*0.90*	*2.50*	
- labour immigrant	15.3%	1.98	*1.39*	*2.80*	
- other non-industrialized	9.6%	1.24	*0.76*	*2.03*	
- other industrialized	11.0%	1.39	*0.71*	*2.74*	
Paternal educational level					
- low	8.9%	1.28	*0.94*	*1.76*	0.007
- medium	9.1%	1.43	*1.06*	*1.94*	
- high	6.4%	1.00	ref		
- unknown	16.9%	2.15	*1.35*	*3.42*	
Family income					
- below poverty	15.9%	1.49	*1.05*	*2.11*	0.017
- above poverty	8.1%	1.00	ref		
- unknown	6.7%	0.78	*0.54*	*1.12*	
Age of the child					
- 14 months	11.4%	1.99	*1.44*	*2.75*	< 0.0001
- 3 years 9 months	8.1%	1.34	*0.94*	*1.91*	
- 5–6 years	8.6%	1.23	*0.92*	*1.64*	
- 7–12 years	7.5%	1.00	ref		

Total	8.7%				

### Professionals' assessment

The prevalence rates of professional-assessed parenting problems during the visit were generally lower than the prevalence rates of any concerns expressed by parents before the visit, see Table 4. Associated with this, many parents reported concerns although the CHP did not identify parenting problems, and the reverse also occurred. However, in a majority of all cases, parent and CHP agreed on the existence of problems, i.e. both responding that there were problems or both that there were no problems. The proportion of children on which agreement existed varied from 73.8% (behavioural problems) to 87.5% (developmental delay). Despite these rather high proportions of agreement, kappas ranged from 0.06 to 0.16 which is rather low [23], though all were highly statistically significant (not shown).

**Table 4:** Concordance between concerns of parents and problems assessed by CHPs on various domains, respectively (n = 4,107) (= 100%))

Domain	Prevalence rates of		Parents have any concerns @		Parent have no concerns @		P #
	Any parental concern	CHP assessed problem	CHP: problems	CHP: no problems	CHP: problems	CHP: no problems	

Behavioural problems	1004 (25.6%)	295 (7.2%)	101 (2.5%)	901 (22.2%)	161 (4.0%)	2891 (71.3%)	
*rate of problems $*			*22.8%*	*15.2%*	*6.8%*	*4.3%*	*< 0.0001*
Emotional problems	622 (15.2%)	265 (6.5%)	82 (2.0%)	539 (13.3%)	213 (5.3%)	3213 (79.4%)	
*rate of problems *^			*47.6%*	*29.4%*	*13.3%*	*5.6%*	*< 0.0001*
Parenting in general	851 (20.8%)	239 (5.9%)	134 (3.3%)	744 (18.3%)	102 (2.5%)	3077 (75.8%)	
Developmental delay	510 (12.5%)	77 (1.9%)	39 (1.0%)	470 (11.6%)	38 (0.9%)	3507 (86.5%)	
Consequences of disease	610 (15.0%)	56 (1.6%)	31 (0.8%	577 (14.3%)	22 (0.5%)	3414 (84.4%)	
*rate of problems*			*83.3%*	*32.6%*	*22.7%*	*7.4%*	*< 0.0001*

@Total numbers of children with parent-reported problems and with CHP assessed problems are slightly lower than columns 2 and 3 (prevalence rates), respectively, because of some omissions in the cross-tabulations. Non-italicised percentages indicate the proportion of all children that is in that category. Italicised percentages indicate the percentage of the children in that category that has a score is in the highest decile on the CBCL/ITSEA.

$ Score in highest decile of externalizing problems on ITSEA (age 14 months) or CBCL (3 years 9 months and over).

^ Score in highest decile of internalizing problems on ITSEA (age 14 months) or CBCL (3 years 9 months and over).

# P value for differences across the categories of concern in rates of parent-reported problems.

& All kappas: p < 0.0001

Regarding behavioural and emotional problems, and regarding the consequences of diseases, we also assessed the prevalence rates of parent-reported problems on the CBCL and ITSEA. We compared the prevalence rates for parental concern that were confirmed or not confirmed by the professional. Table 4 shows that rates of parent-reported problems are highest if parents and professionals share concerns on a specific domain (middle part of Table 4, italicised figures). However, if parents have concerns which are not confirmed by the professional, the rates for problems are still rather high. Similarly, if the CHP has concerns about, in particular, emotional problems that are not shared by the parent, the prevalence rates of CBCL/ITSEA reported problems are also higher, though lower than if a parent has concerns, either shared or not shared by the CHP. Analyses on frequent concerns yielded similar results (not shown). Taking CBCL/ITSEA as the golden standard, the sensitivity of parental concerns to detect an elevated score on these questionnaires is 0.54 and 0.49 for behavioural and emotional problems, respectively. The associated specificity figures are 0.78 and 0.88, respectively.

### Parental reasons for concerns

Finally we assessed the reasons why parents did not seek professional assistance if they had at least one frequent concern (Table 5). The most frequently mentioned reasons were not knowing the appropriate provider of care, confidence that the problem will resolve itself and difficulties experienced in asking help. Relatively many parents (35%) indicated other reasons, but we did not obtain further information on that category. All background characteristics that were associated with the occurrence of frequent concerns, i.e. child age, ethnic background, father's educational level and family income, were also associated with not seeking care for problems (p < 0.0001). Rates of not seeking help were highest for children from former colonial (48%) and labour immigrant families (62%), fathers with low (34%) or unknown (33%) educational level, income below poverty level (38%) or unknown (41%), and ages 7–12 (36%).

**Table 5:** Help-seeking behaviour and reasons for not seeking care among parents with at least one frequent concern regarding parenting (n = 358).

Asked for help	280	78.2%		
Did not ask for help #	78	21.8%		
*Difficult to ask for help*			*12*	*15.4%*
*My partner does not want help*			*7*	*9.0%*
*I do not know the appropriate provider*			*17*	*21.8%*
*I do not like others to know that I need help*			*4*	*5.1%*
*I think that proper care is too expensive*			*1*	*1.3%*
*I have poor previous experiences*			*2*	*2.6%*
*My child does not want help*			*5*	*6.4%*
*Help not needed/resolves itself*			*14*	*17.9%*
*Other reasons*			*28*	*35.9%*
				*115.4%*

# Multiple reasons were allowed to be mentioned.

## Discussion

The results of this study show that half of all parents of children aged 1–12 years in the general population have concerns about the rearing of their child that in their opinion should be discussed with someone outside the family, with concerns on the child's behaviour being most prevalent. Frequent concerns were most likely to occur among parents of young children, of labour-immigrant origin, with low reported income and a medium paternal educational level. Professionals did not confirm most of the parental concerns regarding developmental, behavioural and emotional problems or regarding the consequences of disease. The rates of parent-reported problems were highest for confirmed parental concerns. However, these rates were also high for non-confirmed parental concerns. Lack of knowledge, a low perceived urgency of the problems and difficulties in obtaining care were the main reasons that parents mention for not seeking care.

### Fit with previous studies

We found very high prevalence rates for concerns, showing that having concerns is a rather general aspect of parenting. However, the prevalence rates of frequent concerns in any developmental domain were much lower, 8–12%. Regarding specific domains, these prevalence rates can be compared to those reported by Ford and co-workers who reported on children aged 5–15 [6]. They found parent-report of behavioural and emotional problems of 5.5 and 4.1%, respectively, slightly higher than the rates that we found for frequent concerns in these domains (Figure 1B). Ellingson and co-workers assessed parental concerns regarding behavioural, emotional and social problems among children aged 1–3 years [24]. They report an overall prevalence of 18%, which is in between the prevalence rate of the combination of any and of frequent behavioural and emotional problems in our study for those aged 14 months, and 3 years and 9 months. Finally, Blanchard et al. reported prevalence rates for parental concerns on several more detailed domains than we did, for example gross motor functions in young children and depression and anxiety in older children [25]. For children aged 4–17 months, they report rates of problems of up to about 20% for instance regarding making speech sounds and behaviour. For children aged 6–17 years they report rates of up to 36% for anxiety/depression and 41% for learning difficulties. In conclusion, the questions that we asked seem to elicit a rather wide range of parental concerns.

Frequent parental concerns were more prevalent in families characterized by a number of indicators of societal adversity, such as low income, immigrant status, one-parent families, unemployment, level and young parental age at birth of the first child, with some of these indicators overlapping. Moreover, they were more likely with young children. Ellingson and co-workers reported some similar effects regarding parental concerns in US children aged 1–3, but interestingly they did not find differences between white and non-white families or employment status [24]. An explanation may be that their study was embedded in a cohort study whereas ours was a cross-sectional one embedded in routine care. Deprived groups have generally been shown to be less included in longitudinal research [26], and thus the deprived groups in their study may be a relatively favoured selection from these deprived groups. Being in an adverse situation may in itself lead to more concerns by parents about the future of their child; moreover, parents may have less time or tools for solving adequately the many problems they encounter when rearing their children. Adding a formal assessment of these aspects of parenting to future studies may thus yield a further explanation of these findings.

Parents with frequent *concerns *in the domains behavioural and emotional problems and consequences of diseases much more frequently report *problems in validated questionnaires *in these domains too. This is obviously to be expected, but seems to be forgotten sometimes, causing Glascoe to propose the thesis that parental concerns are a very good screener for assessing child problems. She added that the screening function of concerns becomes better if these concerns are asked for as specifically as possible [3,4]. In this study, as in daily practice, professionals do not always agree with the concerns expressed by parents. However, when they disagree with the frequent concerns that are expressed by parents, rates of parent-reported problems are much increased. The reasons for this discrepancy deserve further study. Are professionals right in not confirming these parental concerns? Or is the discrepancy due to the fact that parents do not raise their concerns during the visit? And if the latter applies, can professionals encourage the expression of these concerns?

Parents in deprived settings, i.e. of non-Dutch background, low family income, etc., were not only more likely to have frequent concerns, but also not to express them. This indicates important barriers in the finding help for these groups. We previously showed that among immigrant groups in the Netherlands, especially among labour immigrant groups i.e. form Morocco and Turkey, the agreement between professional judgement and parental report on the CBCL is much weaker than among Dutch-born parents [22]. Similar cultural barriers may occur in the provision of care regarding developmental problems of children in general. Methods to improve this may be a more regular use of interpreters or bilingual CHPs [27-29], and training CHPs in recognising psychosocial problems in children (and parents) from other cultural backgrounds [29].

Besides solving these cultural barriers, the identification of problems and the recognition of parental concerns by professionals may also be reached by other methods. For instance, regarding parent-reported child behavioural and emotional problems, we previously showed that the degree as to which child health professionals identify them can be improved by structured training [30], and by the use of short questionnaires [31-33]. Evidence on the quality of this identification shows that probably room exists for further improvement [7,8,34]. In addition, other methods may help to improve recognition such as spending more time per visit, and training parents to better express their needs. This potential for improvement is likely to hold for other developmental domains as well. It should be noted though that, like in any type of care, a full agreement between parental concerns and professional recognition is not to be expected.

### Strengths and limitations

The strengths of our study include that it concerns a much larger sample than any previous study on parental concerns and that it covers the entire population of children, including those not asking for care in a setting with high attendance rates. Other strengths include its high response rates and the use of information from both parents and professionals, obtained in routine practice but confidential for each other. A limitation may be that we did not have parent-reported measures on the occurrence of problems for all outcomes on which we asked about concerns. Moreover, our study was cross-sectional, which may limit inferences on causality, but this is unlikely to have affected associations between the occurrence of concerns and background characteristics.

## Conclusion

Our results indicate that parental concerns about various aspects of parenting are highly prevalent. Concerns seem to be rather sensitive measures of children's problems, at least regarding behaviour and emotions. As such, they should be taken very seriously in daily practice, even though the reporting of concerns may be influenced by cultural factors as well. The agreement of child health professionals and parents on these concerns is higher than chance only to a rather limited degree, though. Additional research is needed to determine the reasons why this occurs (including the question whether parents expressed their concerns to the CHP), and whether professionals are right in doing so. Use of very explicit interview schemes such as the Parents' Evaluation of Developmental Status as developed by Glascoe [3]. in particular deserves further study regarding this. The size of the problem as indicated shows that it deserves much more attention in both research and routine care, including the reasons why parents do not seek care even if they have frequent concerns.

## Competing interests

The author(s) declares that they have no competing interests.

## Appendix: Questions to parents about their concerns

Have you had concerns regarding parenting, the behaviour or development of your child during the past 12 months that have caused you to need professional help or advice from someone outside the own family (please also answer this question if you have already received help)

Concerns during the past 12 months (No concerns Some concerns Frequent concerns)

-on parenting in general

-on developmental delay

-on behavioural problems (e.g. sleeping, eating)

-on emotional problems (e.g. being afraid or upset)

-on the consequences of disease of the child

-on the contacts of the child with other children

## Authors' contributions

SAR had the original idea for the project, wrote the study protocol, and coordinated the study. All authors discussed the protocol and formulated the final design. MRC and CHW supervised the data collection for the study. SAR did the statistical analyses, which were discussed by all authors. SAR wrote the final manuscript, which was discussed, edited and revised by all authors. All authors read and approved the final manuscript.

## Pre-publication history

The pre-publication history for this paper can be accessed here:


